# MiR-192 attenuates high glucose-induced pyroptosis in retinal pigment epithelial cells via inflammasome modulation

**DOI:** 10.1080/21655979.2022.2044734

**Published:** 2022-04-20

**Authors:** Cao Gu, Hongjun Zhang, Qing Li, Shaofei Zhao, Yu Gao

**Affiliations:** a Department of Ophthalmology, Changhai Hospital, First Affiliated Hospital of Naval Medical University (Second Military Medical University), Shanghai, China; b Department of Ophthalmology, Minhang Branch of Zhongshan Hospital Affiliated to Fudan University, Shanghai, China

**Keywords:** MiR-192, diabetic retinopathy, pyroptosis, retinal pigment epithelial cell, fto

## Abstract

Diabetic retinopathy is one of the most characteristic complications of diabetes mellitus, and pyroptosis plays acrucial role in the onset and development of diabetic retinopathy. Although microRNA-192 (miR-192) has been demonstrated to be involved in diabetic retinopathy progression, to the best of our knowledge, its potential and mechanism in cell pyroptosis in diabetic retinopathy have not been studied. The present study demonstrated that high glucose (HG) contributes to the pyroptosis of retinal pigment epithelial (RPE) cells in a dose-dependent manner. The results revealed that miR-192 was weakly expressed in HG-induced RPE cells. Furthermore, overexpression of miR-192 abrogated the role of HG in RPE cell pyroptosis. Based on the bioinformatics analysis, a dual-luciferase reporter assay, and an RNA pull-down assay, FTO α-ketoglutarate-dependent dioxygenase (FTO) was demonstrated to be a direct target of miR-192. Additionally, upregulation of FTO abolished the effects of miR-192 on RPE cells treated with HG. Nucleotide-binding domain leucine-rich repeat family protein 3 (NLRP3) inflammasome activation is vital for cell pyroptosis, and FTO functions as a pivotal modulator in the N^6^-methyladenosine modifications of various genes. Mechanistically, FTO enhanced NLRP3 expression by facilitating demethylation of NLRP3. In conclusion, the present results demonstrate that miR-192 represses RPE cell pyroptosis triggered by HG via regulation of the FTO/NLRP3 signaling pathway.

## GRAPHICAL ABSTRACT

**Figure:**
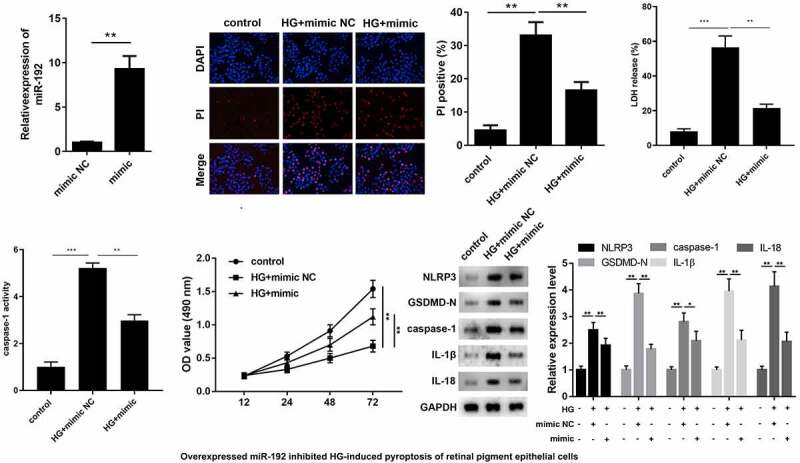

## Highlights

- HG-treatment induced the pyroptosis of retinal pigment epithelial cells.
- Upregulated miR-192 suppressed the pyroptosis.
- miR-192 participated in the development of diabetic retinopathy via binding to FTO.
- FTO regulated the m6A modification of NLRP3.

## 1. Introduction

The incidence of diabetes mellitus is increasing worldwide due to the economic development and changes in lifestyle and dietary habits [1,2]. Diabetic retinopathy is one of the most severe complications of diabetes mellitus and remains the leading contributor to incurable vision loss among working age adults [3]. Diabetic retinopathy is considered as a progressive retinal microvascular disease resulting from hyperglycemia, which features pathological manifestations, such as retinal inflammation, microaneurysm formation, dysfunction of pericytes and endothelial cells, increased vascular permeability, and retinal neovascularization [4–8]. The high worldwide prevalence of diabetic retinopathy among diabetic patients poses a severe threat to public health [9,10]. Despite numerous studies attempting to elucidate the pathogenesis of diabetic retinopathy, its complicated molecular mechanism remains elusive. Therefore, it is urgent to identify potential indicators in the intervention and therapy of this disease.

Inflammatory cell necrosis, also known as pyroptosis, is an emerging type of cell death that has attracted increased attention from medical investigators [11–14]. The NLRP3 inflammasome is responsible for the processing and maturation of caspase-1, and cleaved caspase-1 activates pre-IL-1β and pre-IL-18 and promotes the release of IL-1β and IL-18 [15,16]. The N-terminus of gasdermin (GSDMD-N) is released from GSDMD, which is an essential component of the inflammasome, and serves an ultimate direct executive role in pyroptosis [17,18]. Accumulating evidence has demonstrated the involvement of pyroptosis in the pathological progression of various diseases, including diabetic retinopathy [19–22]. Accordingly, it is necessary to identify the underlying regulators of cell pyroptosis, which may provide novel insights into the treatment of diabetic retinopathy.

The retinal pigment epithelium (RPE), as the major component of the blood-retinal barrier, is vital for the maintenance of retinal function and immune homeostasis [23]. RPE cell dysfunction is strongly associated with the occurrence and development of retinal degenerative disease [24]. Previous studies have indicated that high glucose can induced pyroptosis of RPE cells [25].

MicroRNAs (miRNAs/miRs) are a family of non-coding single-stranded RNAs, which are 21–23 nucleotides long and serve as core regulators in different biological activities, including cell proliferation, apoptosis, pyroptosis, migration and metabolism [26]. Numerous studies have revealed abnormal expression of miRNAs in the progression of diabetic retinopathy [27–30]. As one of them, miR-192 locates in chr19: 6,314,874–6,314,962 with a mature length of 21 nucleotides. Importantly, miR-192 has been reported to be downregulated in the retinas of diabetic rats, and gene ontology analysis suggests that miR-192 is associated with apoptosis [31]. In addition, adipose mesenchymal stem cell-secreted extracellular vesicles containing microRNA-192 delay diabetic retinopathy by targeting ITGA1 [32]. However, the function and molecular mechanism of miR-192 in the pyroptosis of RPE cells during diabetic retinopathy development remains elusive.

Therefore, the aim of the present study was to shed light on the potential role of miR-192 in modulating RPE cell pyroptosis and. We hypothesized that miR-192 may play a crucial role in the development of diabetic retinopathy.

## 2. Materials and Methods

### 2.1 Cell culture and treatment

Human RPE cell line ARPE-19 was supplied by American Type Culture Collection (ATCC, USA) and grown in low-glucose DMEM medium (Gibico, USA) with 10% FBS (Gibico) and 1% penicillin-streptomycin (Gibico). RPE cells were cultivated at 37°C in a humidified incubator with 5% CO_2_. For high-glucose stimulation, ARPE-19 cells were maintained under different concentrations of high-glucose (10, 50, and 100 nM) for 48 h. 30 mM of mannitol was used to treat cells in the mannitol control group.

### 2.2 Cell transfection

To construct the FTO α-ketoglutarate-dependent dioxygenase (FTO)-expressing vectors, the sequences of FTO were inserted into pcDNA3.1 plasmids. For overexpression of FTO, the pcDNA3.1/FTO vector synthesized by Sangon Biotech Co., Ltd. was used with an empty plasmid as the negative control (NC). ARPE-19 cells were transfected with the indicated oligonucleotides (50 nM) and plasmids (50 nM) using Lipofectamine® 2000 (2 µl, Invitrogen; Thermo Fisher Scientific, Inc.), and all procedures complied with the manufacturer’s protocols.

### 2.3 Cell proliferation assays

The viability of RPE cells was measured using a 3-(4,5-Dimethylthiazolyl-2)-2,5-Diphenyl Tetrazolium bromide (MTT) kit according to the manufacturer’s instructions. Following different treatments, RPE cells were seeded into 96-well plates at a density of 2 × 10^3^ cells/well. At 12, 24, 48, and 72 h after culture, RPE cells were incubated with MTT solution for another 4 h at 37°C. Subsequently, 100 µl DMSO was added to each well after the medium was discarded. Absorbance was measured at 490 nm using a microplate reader (Epson LX-800; Epson America, Inc.).

### 2.4 Cell death assay

After transfection, cells were lyzed, The supernatants were collected and analyzed for Lactate dehydrogenase (LDH) level determined with CytoTox 96 LDH Release Kit (Promega).

### 2.5 Pore formation assay

PI staining was performed to analyze pore formation as previously described [33]. RPE cells were inoculated into 24-well plates at a concentration of 1 × 10^5^ cells per well and treated with 6 μl/ml PI reagent for 48 h. RPE cells containing membrane pores were stained with PI, and DAPI was used to stain the cell nuclei. The uptake of PI was adopted to evaluate the formation of pores, and the results were represented as the percentage of PI-positive cells using ImageJ software (National Institutes of Health).

### 2.6 Measurement of caspase-1 activity

Caspase-1 activity was detected as previously described [34]. Briefly, total protein were collected from cells and concentrated using a BCA kit (Bio-Rad, CA, USA). The absorbance values at 405 nm were detected by a spectrophotometer (BioTek, VT Lab, USA). The Caspase-1 activity was calculated using a Caspase-1 activity assay kit.

### 2.7 Reverse transcription quantitative PCR (RT-qPCR)

Total RNA was extracted from RPE cells using TRIzol® reagent (Invitrogen; Thermo Fisher Scientific, Inc.) according to the manufacturer’s instructions. Subsequently, cDNA synthesis was performed using a first-strand cDNA synthesis kit (Takara Biotechnology Co., Ltd.). Gene expression was detected using a Real-Time PCR system (Applied Biosystems; Thermo Fisher Scientific, Inc.) with SYBR Green PCR master mix (Thermo Fisher Scientific, Inc.). The 2^−ΔΔCq^ method [35] was used to calculate the experimental results. U6 and GAPDH were used as loading controls for normalization. The sequences of the primers used in this study were as followed: miR-192: Forward 5’-GCGGCGGCTGACCTATGAATTG-3’ and reverse: 5’-ATCCAGTGCAGGGTCCGAGG-3’; FTO: Forward 5’-ACTTGGCTCCCTTATCTGACC-3’ and reverse: 5’-TGTGCAGTGTGAGAAAGGCTT-3’; NLRP3: Forward 5’-GATCTTCGCTGCGATCAACAG-3’ and reverse: 5’-CGTGCATTATCTGAACCCCAC-3’; GAPDH: Forward 5’-CTGGGCTACACTGAGCACC-3’ and reverse: 5’-AAGTGGTCGTTGAGGGCAATG-3’ U6: Forward 5’-GATTATCGGGACCATTCCACTG-3’ and reverse: 5’-GATCTGGTTCCCAATGACTGTG-3’.

### 2.8 Western blotting

Equivalent samples were separated via 10% SDS-PAGE, followed by transfer to PVDF membranes and blocking in 5% skimmed milk. Subsequently, the membranes were probed with primary antibodies against NLRP3 (Abcam), GSDMD-N (Abcam), caspase-1 (Cell Signaling Technology, Inc.), IL-1β (Abcam), IL-18 (Abcam), FTO (Cell Signaling Technology, Inc.), or GAPDH (Cell Signaling Technology, Inc.) at 4°C overnight, followed by treatment with the appropriate secondary antibodies. The protein bands were visualized using achemiluminescence system kit (Thermo Fisher Scientific, Inc.). The intensity of the bands was analyzed using ImageJ software. GAPDH served as an internal reference.

### 2.9 RNA pull down assay

Biotin-labeled miR-192 was obtained using an RNA 3ʹEnd Desthiobiotinylation Kit (Pierce; Thermo Fisher Scientific, Inc.) according to the manufacturer’s protocol. Collected RPE cells were lysed in lysis buffer (Pierce; Thermo Fisher Scientific, Inc.) and incubated with biotinylated miR-192 or a NC. Subsequently, streptavidin magnetic beads were added to the mixture. Following overnight incubation, the RNAs were eluted from the beads using washing buffer and subjected to RT-qPCR analysis.

### 2.10 Dual-luciferase reporter gene assays

To synthesize wild-type (WT) or mutant-type (Mut) FTO constructs, the 3’-untranslated regions of FTO containing WT or Mut binding sequences for miR-192 were ligated into the luciferase reporter gene plasmid pMIR-REPORT (Thermo Fisher Scientific, Inc.). RPE cells were co-transfected with the described plasmids and miR-192 mimic or NC oligonucleotides using Lipofectamine® 2000 according to the manufacturer’s protocols. Luciferase activity was quantified using a dual-luciferase reporter assay system (Promega Corporation) according to the manufacturer’s protocol.

### 2.11 N^6^-methyladenosine (m^6^A) quantification assay

The m^6^A levels in total RNA from RPE cells were examined using the EpiQuik m^6^A RNA Methylation Quantification Kit (EpiGentek Group, Inc.) according to the manufacturer’s instructions.

### 2.12 Methylated RNA immunoprecipitation

Total RNA extracted from the RPE cells was purified and sonicated into slices. The RNA fragments were immunoprecipitated using an anti-m^6^A antibody in IP buffer containing protein A/G magnetic beads. After elution and purification, the abundance of NLRP3 in the immunoprecipitated RNA was measured by RT-qPCR.

### 2.13 Statistical analysis

Each independent experiment was performed in triplicate. Data processing was performed using SPSS software (version 19.0; SPSS Inc., USA). The difference was analyzed using student t test and ANOVA. Statistical significance was set at P < 0.05.

## 3. Results

This study investigated the potential roles of miR-192 in diabetic retinopathy. miR-192 suppressed the pyroptosis of RPE via targeting FTO. Moreover, FTO regulated m6A modification of NLRP3.

### 3.1 High glucose (HG) inhibits cell proliferation and activates pyroptosis in RPE cells

**Figure 1.**
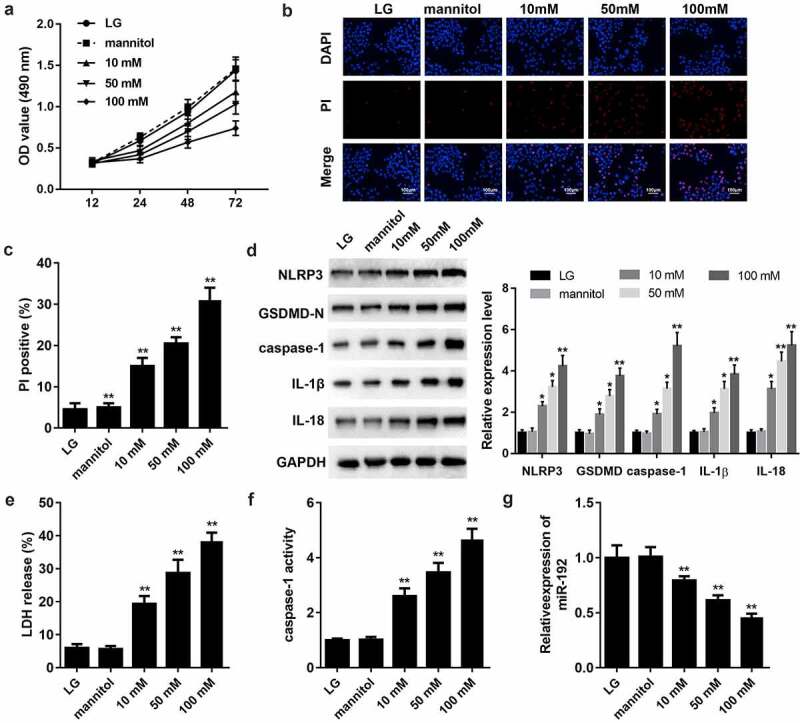
HG treatment inhibits cell proliferation and activates the pyroptosis of RPE cells. ARPE-19 cells were treated with 0, 10, 50, or 100 mM glucose for 48 h. (a) RPE cell viability was measured using an MTT assay. (b, c) PI staining was performed to determine cell death. (d) Western blot analysis of the expression levels of hallmark proteins (nucleotide-binding domain leucine-rich repeats family protein 3, caspase-1, N-terminal of gasdermin, IL-1β, and IL-18) in cell pyroptosis. (e) The release of LDH. (f) The activity of caspase-1. (g) Reverse transcription-quantitative PCR detection of miR-192 expression in ARPE-19 cells following HG treatment. Experimental data are presented as the mean ± SD (n = 6). *P < 0.05, **P < 0.01. RPE cell, retinal pigment epithelial cell; HG, high glucose.

To establish an invitro model of diabetic retinopathy, RPE cells were treated with LG, mannitol or HG. Cell viability of RPE cells was decreased by HG treatment (Fig. 1A). Subsequently, the effects of HG on pyroptosis were assessed using a pore formation assay and characteristic factor-level detection. As shown in Fig. 1BandC, HG treatment increased PI positive cells in a dose-dependent manner. The protein expression levels of NLRP3, GSDMD-N, caspase-1, IL-1β, and IL-18 increased with increasing glucose concentrations (Fig. 1D). HG treatment increased the release of LDH (Fig. 1E) and caspase-1 activity (Fig. 1F). Additionally, RT-qPCR analysis suggested that miR-192 expression was markedly downregulated in HG-induced RPE cells (Fig. 1G). Therefore, HG may trigger RPE cell pyroptosis.

### 3.2 miR-192 restrains HG-induced pyroptosis in RPE cells

**Figure 2.**
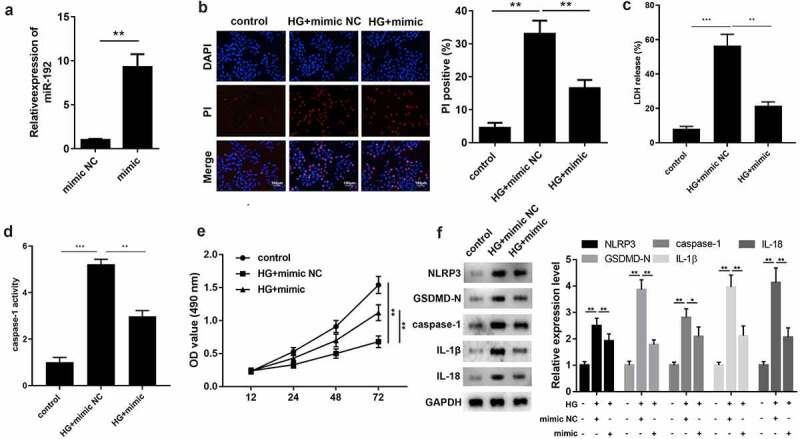
miR-192 restrains HG-induced pyroptosis in RPE cells. ARPE-19 cells were transfected with miR-192 mimic or NC oligonucleotides after administration of HG. (a) Transfection efficiency of miR-192 overexpression was confirmed using reverse transcription-quantitative PCR. (b) PI staining was performed to determine cell death. (c) The release of LDH. (d) The activity of caspase-1. (e) MTT analysis was employed to estimate the proliferation of transfected ARPE-19 cells. (f) Western blot analysis of the expression levels of pyroptosis-associated proteins. Experimental data are presented as the mean ± SD (n = 6). **P < 0.01, ***P < 0.001. miR-192, microRNA-192; RPE cell, retinal pigment epithelial cell; HG, high glucose.

To identify the function of miR-192 in RPE cell pyroptosis, miR-192 was overexpressed in RPE cells by transfection with an miR-192 mimic, and the results of RT-qPCR demonstrated the transfection efficiency (Fig. 2A). Overexpresion of miR-192 significantly decreased PI positive cells (Fig. 2B), the release of LDH (Fig. 2C), and caspase-1 activity (Fig. 2D). In addition, enhanced miR-192 expression abolished the effects of HG treatment on RPE cell proliferation (Fig. 2E). Western blotting demonstrated that the decreased expression levels of NLRP3, GSDMD-N, caspase-1, IL-1β, and IL-18 in HG-induced RPE cells was restored by upregulation of miR-192 (Fig. 2F). Taken together, these findings demonstrate that miR-192 ameliorates the pyroptosis of RPE cells resulting from HG treatment.

### 3.3. miR-192 negatively modulates FTO expression

**Figure 3.**
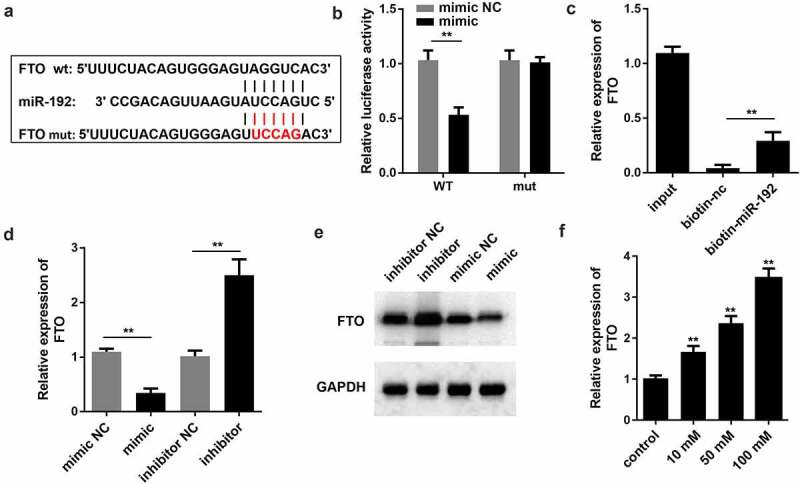
miR-192 negatively modulates the expression levels of FTO. (a) miR-192 binding sites in the 3’ untranslated region of FTO as predicted by TargetScan. (b and c) Interaction of FTO with miR-192 was validated by a dual-luciferase reporter assay and RNA pull down. (d) Reverse transcription-quantitative PCR was conducted to evaluate the effects of miR-192 on FTO expression. (e) Western blot was applied to determine the protein expression of FTO. (f) Reverse transcription-quantitative PCR was used to evaluate the FTO expression after high glucose treatment. Experimental data are presented as the mean ± SD (n = 4). **P < 0.01 vs control. miR-192, microRNA-192; FTO, FTO α-ketoglutarate dependent dioxygenase.

To explore the regulatory mechanism of miR-192, the present study aimed to identify the potential downstream targets of miR-192. By browsing the TargetScan website (http://www.targetscan.org/vert_72/), we found that there were predicted binding sites between miR-192 and FTO (Fig. 3A). Subsequently, a dual-luciferase reporter gene assay was performed to verify this association. As shown in Fig. 3B, the miR-192 mimic only decreased the luciferase activity of FTO-WT, while no significant alteration was observed for the mutant form of FTO. The RNA pull-down assay demonstrated that FTO expression was abundant in precipitates pulled down by miR-192, which further validated that miR-192 directly bound to FTO (Fig. 3C). RT-qPCR and Western blot revealed that the mRNA and protein expression of FTO were decreased by miR-192 mimic, whereas suppression of miR-192 led to an increase in FTO expression (Fig. 3DandE). In addition, miR-192 FTO expression was upregulated by HG treatment (Fig. 3F). Overall, these results indicated that FTO is a downstream target of miR-192.

### 3.3 Overexpression of FTO counteracts the regulatory role of miR-192 in RPE cell pyroptosis

**Figure 4.**
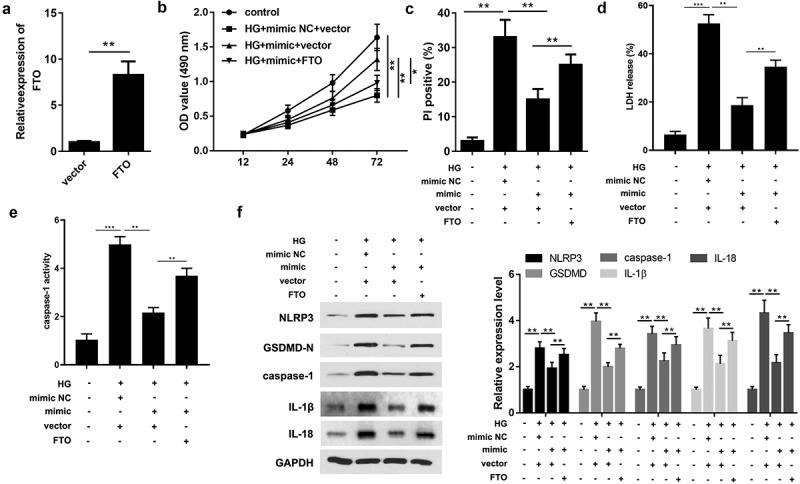
Overexpression of FTO counteracts the regulatory role of miR-192 in retinal pigment epithelial cell pyroptosis. (a) Reverse transcription-quantitative PCR analysis of the transfection efficiency of FTO overexpression. (b) MTT detection of ARPE-19 cell viability under different treatment conditions. (c) Cell death detected by PI staining. (d) The release of LDH. (e) The activity of caspase-1. (f) The protein expression detected using Western blot. Experimental data are presented as the mean ± SD (n = 4). **P < 0.01, ***P < 0.001. FTO, FTO α-ketoglutarate dependent dioxygenase; miR-192, microRNA-192; HG, high glucose; NC, negative control.

Based on the aforementioned outcomes, it is reasonable to infer that miR-192 acts as a regulator of HG-induced RPE cell proliferation and pyroptosis by repressing FTO. Therefore, FTO expression was upregulated in further experiments (Fig. 4A). The MTT assay showed that overexpression of FTO abrogated the protective function of miR-192 in the proliferation of RPE cells treated with HG (Fig. 4B). Furthermore, FTO significantly increased PI positive cells (Fig. 4C), the release of LDH (Fig. 4D) and caspase-1 activity (Fig. 4E). Consistently, upregulation of FTO abolished the marked decrease in the expression levels of proteins associated with pyroptosis caused by miR-192 overexpression in RPE cells treated with HG (Fig. 4F). This suggests that the role of miR-192 in HG-induced pyroptosis of RPE cells is mediated by FTO.

### 3.4 FTO regulates NLRP3 expression in an m^6^A-dependent manner

**Figure 5.**
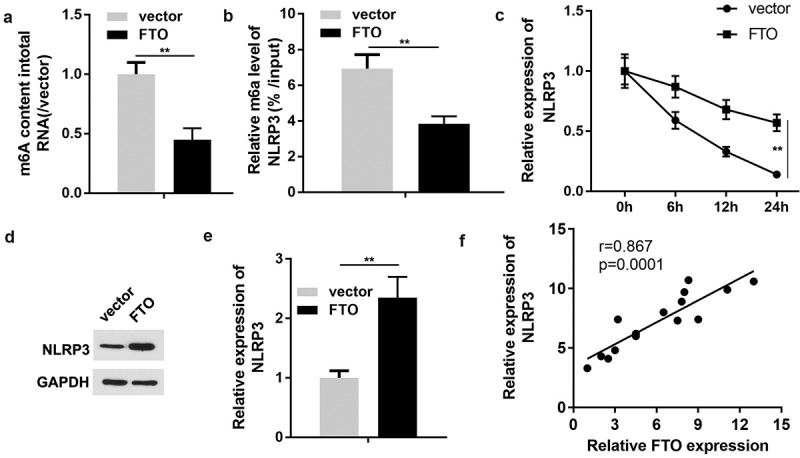
FTO regulates NLRP3 expression in a m^6^A-dependent manner. (a) Quantification of m^6^A levels in transfected ARPE-19 cells. (b) Methylated RNA immunoprecipitation was conducted to determine the enrichment of NLRP3 mRNA by utilizing m^6^A-specific antibody. (c) The mRNA stability detected using PCR. (d, e) Western blot analysis of NLRP3 expression in ARPE-19 cells transfected with FTO-expressing vector or negative control empty vector. (f) Pearson correlation analysis of the association between FTO and NLRP3. Experimental data are presented as the mean ± SD (n = 4). **P < 0.01. FTO, FTO α-ketoglutarate dependent dioxygenase; NLRP3, nucleotide-binding domain leucine-rich repeats family protein 3; m^6^A, N^6^-methyladenosine.

Since FTO serves as a modulator of m^6^A modification, the present study ultimately investigated the association between FTO and the characteristic markers of pyroptosis. The results of RNA m^6^A quantification revealed that enhanced FTO expression was associated with a reduction in m^6^A levels (Fig. 5A). Additionally, the Me-RIP assay revealed that ectopic expression of FTO markedly decreased the m^6^A modification levels of NLRP3 (Fig. 5B). qPCR analysis revealed that FTO overexpression promoted the mRNA stability of NLRP3 (Fig. 5C). Western blotting indicated that NLRP3 expression was augmented by the upregulation of FTO (Fig. 5DandE). Pearson’s correlation analysis suggested that FTO expression was positively associated with NLRP3 expression (Fig. 5F). Based on these results, we hypothesized that miR-192 suppressed RPE cell pyroptosis induced by HG treatment via regulation of the FTO- m^6^A /NLRP3 axis.

## 4. Discussion

Diabetic retinopathy ranks fifth among the leading causes of blindness worldwide, severely compromises the quality of life of patients, and represents a serious health threat [36,37]. There are multiple therapeutic options that have achieved moderate clinical efficacy, such as steroids; however, treatments that can completely mitigate clinical progression and reverse retinal damage have not been developed to date [38–40]. An increasing number of studies have demonstrated that persistent hyperglycemia induced by diabetes can provoke RPE cell death, which promotes the development of diabetic retinopathy [41–44]. Considering the critical role of RPE cells in diabetic retinopathy progression, HG-induced RPE cells have been extensively employed as an ideal model for in vitro experiments of diabetic retinopathy [45–47]. Therefore, the present study aimed to characterize the regulatory factors in the function of RPE cells treated with HG and to comprehensively elucidate the molecular mechanisms governing diabetic retinopathy pathophysiology.

Dysregulated miRNAs have been demonstrated to be involved in the pathogenesis of diabetes and its complications [48,49]. Furthermore, differentially expressed miRNAs function as diagnostic biomarkers for diabetic retinopathy [29,50]. Numerous studies have investigated the role of miR-192 in various diabetic complications [51,52]. In diabetic nephropathy, the expression levels of miR-192 are closely associated with disease progression, suggesting its potential as an indicator in the diagnosis and determination of diabetic nephropathy [53–56]. Notably, low expression levels of miR-192 in mouse models of diabetic retinopathy have been previously confirmed [31]. Furthermore, miR-192 ameliorated the inflammatory response in HG-induced RPE cells [33]. However, the involvement of miR-192 in cell pyroptosis during the pathogenesis of diabetic retinopathy remains unclear. To the best of our knowledge, the present study is the first to demonstrate that HG treatment restricts cell proliferation and contributes to the pyroptosis of RPE cells. In addition, miR-192 was weakly expressed in the RPE cells induced by HG treatment. Subsequently, overexpression of miR-192 reversed the effects of HG treatment on RPE cell pyroptosis.

Numerous studies have demonstrated that m^6^A modification, as the most frequent post-transcriptional modification, is implicated in the physiological and pathological processes of various diseases, including diabetes [57]. The m^6^A demethylase FTO is recognized as an m^6^A modification ‘eraser’ and plays a vital role in the regulation of m^6^A modifications [58,59]. FTO is located on chr16:53,701,692–5,415,512 with a size of approximately 58 kDa. It is a nuclear protein of the AlkB-related non-heme iron and 2-oxoglutarate-dependent oxygenase superfamily. As an RNA demethylase, FTO mediates oxidative demethylation of different RNA species, such as mRNAs, tRNAs, and snRNAs, and acts as a regulator of fat mass, adipogenesis, and energy homeostasis [60]. Although the association between FTO expression and diabetes has been affirmed [61], the potential of FTO in diabetic retinopathy remains to be elucidated. The present study revealed that FTO is a downstream target of miR-192. Furthermore, miR-192 protects against RPE cell death caused by HG treatment by suppressing FTO expression. Notably, the present results revealed that FTO facilitated m^6^A modification-mediated NLRP3 activation, providing evidence supporting the hypothesis that miR-192 alleviates HG-induced pyroptosis of RPE cells in an m^6^A-dependent manner. However, as we know, m6A-dependent regulation is based on RNA methylases, demethylases, and readers. The current study did not determine if the reader can influence the stability or translation of the target RNA. While this is a limitation of the present study, future work will be completed to investigate this.

## Conclusion

Overall, the present study focused on investigating the role of miR-192 in pyroptosis in diabetic retinopathy and elucidated its regulatory molecular mechanism. The findings revealed that miR-192 delayed the damage to RPE cells triggered by HG treatment by targeting FTO, which improved diabetic retinopathy therapy. Further experiments are required to validate whether the function of miR-192 in HG-induced RPE cells depends on the m^6^A-regulated NLRP3 activation signaling pathway.
